# Characterization of Pelvic Floor Activity in Healthy Subjects and with Chronic Pelvic Pain: Diagnostic Potential of Surface Electromyography

**DOI:** 10.3390/s21062225

**Published:** 2021-03-23

**Authors:** Monica Albaladejo-Belmonte, Marta Tarazona-Motes, Francisco J. Nohales-Alfonso, Maria De-Arriba, Jose Alberola-Rubio, Javier Garcia-Casado

**Affiliations:** 1 Centro de Investigación e Innovación en Bioingeniería, Universitat Politècnica de València, 46022 Valencia, Spain; moalbel@ci2b.upv.es (M.A.-B.); jgarciac@ci2b.upv.es (J.G.-C.); 2 Servicio de Ginecología y Obstetricia, Hospital Politècnic i Universitari La Fe, 46026 Valencia, Spain; tarazona_marmot@gva.es (M.T.-M.); franoal@uv.es (F.J.N.-A.); dearriba_margar@gva.es (M.D.-A.); 3 Unidad de Bioelectrónica, Procesamiento de señales y Algoritmia, Instituto de Investigación Sanitaria La Fe, 46026 Valencia, Spain

**Keywords:** chronic pelvic pain, myofascial pain syndrome, deep dyspareunia, pelvic floor musculature, surface electromyography, diagnosis

## Abstract

Chronic pelvic pain (CPP) is a highly disabling disorder in women usually associated with hypertonic dysfunction of the pelvic floor musculature (PFM). The literature on the subject is not conclusive about the diagnostic potential of surface electromyography (sEMG), which could be due to poor signal characterization. In this study, we characterized the PFM activity of three groups of 24 subjects each: CPP patients with deep dyspareunia associated with a myofascial syndrome (CPP group), healthy women over 35 and/or parous (>35/P group, i.e., CPP counterparts) and under 35 and nulliparous (<35&NP). sEMG signals of the right and left PFM were recorded during contractions and relaxations. The signals were characterized by their root mean square (RMS), median frequency (MDF), Dimitrov index (DI), sample entropy (SampEn), and cross-correlation (CC). The PFM activity showed a higher power (>RMS), a predominance of low-frequency components (<MDF, >DI), greater complexity (>SampEn) and lower synchronization on the same side (<CC) in CPP patients, with more significant differences in the >35/P group. The same trend in differences was found between healthy women (<35&NP vs. >35/P) associated with aging and parity. These results show that sEMG can reveal alterations in PFM electrophysiology and provide clinicians with objective information for CPP diagnosis.

## 1. Introduction

Chronic pelvic pain (CPP) is a complex clinical condition characterized by persistent or recurrent pelvic pain for longer than six months with no proven infection or obvious pathology [1]. One of the most common syptoms associated with its appearance is dyspareunia, defined as recurrent or persistent genital pain during sexual intercourse [2]. In women, dyspareunia is labeled as “deep” or “superficial”, depending on whether it appears on vaginal introitus at the beginning of the penetration or within the pelvis when penetration is deeper, respectively [3]. Dyspareunia has been estimated to affect between 8% and 21.1% of the worldwide population, a value that is highly variable among countries [4]. For instance, 7.5% of sexually active women in Britain suffer from dyspareunia [5], while the prevalence in the United States ranges from 17% to 19% [6]. Some studies indicate that up to 61% of women experience pain during intercourse at least once in their lives [7]. Although different authors have addressed the economic impact of CPP [8,9], this assessment has not been specifically performed in the case of dyspareunia as the main CPP manifestation. However, the total cost of vulvar pain, which is intimately associated with superficial dyspareunia, ranges from $31 to $72 billion per year in the US [10], indicating the high economic burden of CPP associated with dyspareunia. As for its social impact, CPP associated with dyspareunia or other disorders can be highly disabling and have a serious impact on women’s lives. It can significantly alter social activities, psychical health, professional life, finances, and sexual relationships [11].

CPP and other clinical conditions of the pelvic floor (pelvic organ prolapse, urinary/fecal incontinence, etc.) are closely associated with dysfunction of the pelvic floor musculature (PFM) [12], so that surface electromyography is considered to be a useful tool to assess patients’ clinical condition [13,14]. The surface electromyogram (sEMG) is the electric signal recorded by means of electrodes attached to the surface of the skin or inside an anatomical opening produced by the sum of the extracellular potentials from the active muscle fibers beneath the electrodes [15]. Various authors have reported overactivity of the PFM in CPP patients with dyspareunia, which suggests the possible role of the PFM in this disorder [13]. Different researchers have attempted to determine and fulfill the diagnostic potential of sEMG in this clinical area. Whereas some of them reported promising results [16,17,18], Engman et al. [19] concluded that sEMG recordings were not suitable to differentiate between patients with superficial dyspareunia and assymptomatic women. In all of these studies, the analysis was focused on changes in the amplitude of the sEMG signal according to its averaged rectified value or root mean square, among others. The assessment of other sEMG featuress like spectral or non-linear values has not been studied, despite the strong evidence in the literature on their value in providing relevant information on the electrophysiologic state of the muscle [20,21,22]. In the field of chronic pain, Sung et al. [23] reported that patients with persistent low back pain presented myoelectrical activity with a significantly lower level of complexity than healthy subjects.

Given its potential to characterize PFM activity, sEMG has not been exclusively devoted to the study of the PFM in dysfunctional conditions but also to different physiological circumstances. Special attention has been paid to women’s demographic and obstetric characteristics. Different studies have reported a negative correlation between women’s age and their PFM activity [24,25], as well as a decrease of such activity with pregnancy and parity [24,26,27]. Some other scenarios in which differences in voluntary and involuntary PFM activity have been assessed are the performance of training programs [28,29], the position of the different lower body segments [30,31,32] and changes in sexual hormone levels throughout the menstrual cycle [33].

The purpose of the present study was (1) to characterize the PFM activity of CPP patients whose main presentation is deep dyspareunia associated with a myofascial pain syndrome and that of healthy women with similar and different demographic and obstetric characteristics to a CPP patients’ database, and (2) to assess the diagnostic value of sEMG in CPP in which musculoskeletal pelvic pain is a main component. To this end, we recorded the sEMG signal from the left and right sides of the pelvic floor with adhesive electrodes and characterized the power, spectral content and complexity of the signal during PFM maximum voluntary contractions and relaxation, as well as interactions between different regions on the same PFM side. The results obtained prove that sEMG can reveal alterations in the PFM electrophysiology associated with different processes like aging, parity, or pain and provide clinicians with objective information that can help them to better evaluate CPP patients’ condition, thus allowing more efficient management of this complex syndrome.

## 2. Materials and Methods

### 2.1. Database Composition

This study is included in the clinical trial “Electromyographic Study for the Help and Guidance of Botulinum neurotoxin A Administration in the Treatment of Chronic Pelvic Floor Pain (SEMG)” (Clinical Trials: NCT03715777) conducted at the Hospital Universitari i Politècnic La Fe (Valencia, Spain), a prospective, minimally invasive, non-masked and non-randomized phase III clinical trial that meets the Helsinki Declaration. Female subjects with different demographic, obstetric, and clinical characteristics were recruited: healthy women over 35 years old and/or with previous pregnancies (group >35/P), under 35 years old with no previous pregnancies (group <35&NP), and women with CPP for more than 6 months whose main presentation was deep dyspareunia associated with a myofascial pain syndrome (group CPP). The subjects from the last group met some supplementary inclusion criteria: age of majority (18 years), no active pelvic infections and no general malignant, pelvic or psychiatric diseases. A total of 72 subjects were recruited, 24 per group, who provided their informed consent after they were acquainted with the nature of the study.

The main demographic and clinical characteristics of the three groups are shown in Table 1. For each of them, statistically significant differences between each pair of groups (significance level: 5%) have been indicated with a different symbol. Comparisons were made according to the Kruskal–Wallis test and the Bonferroni correction due to non-normality of data, checked by the Kolmogorov–Smirnov test. In the case of menopause, Fisher’s exact test was performed. No significant differences were found between CPP and >35/P groups in any characteristic. Age, parity and the prevalence of menopause were significantly higher in these groups than in the <35&NP, except for menopause in the case of >35/P vs. <35&NP, as could be expected according to the criteria followed to build the group. In the CPP and >35/P groups, mean age was over 40, mean number of deliveries was 1.5 and some women had gone through menopause, while in the <35&NP group mean age was under 30, all the subjects were nulliparous and none had reached the menopause. In this group, weight was lower and height was higher than in the CPP (significantly only for weight) and >35/P (significant only for height), also an indirect consequence of the recruitment protocol.

### 2.2. sEMG Signal Recording

**Figure 1:**
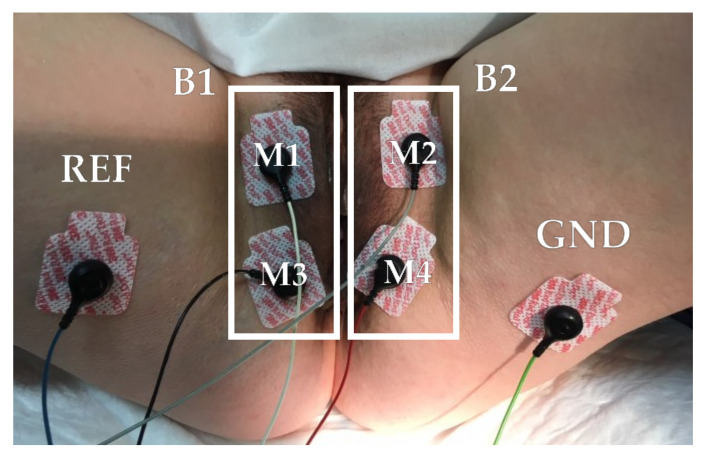
Electrodes location for the surface electromyogram (sEMG) signal recording.

Patients remained in a dorsal lithotomy position during the recording of the sEMG signal. Surface electromyography recordings were performed with adhesive electrodes over the perineum to assess PFM myoelectrical activity. They were chosen over intravaginal probes since such probes can be uncomfortable and provoke pain during their insertion, specially in patients that suffer from genital pain. This could hamper the acceptance of sEMG techniques by patients and thus its use in the clinical practice. Four disposable Ag/AgCl electrodes (Red Dot 2660-5, 3M, St. Paul, MN, USA) were attached to both labia majora and two additional electrodes (ground: GND, reference: REF) were placed on both iliac spines, as Figure 1 displays. Four monopolar sEMG signals were recorded (M1, M2, M3, M4) with a multipurpose biomedical signal amplifier (Grass 15LT+4 Grass 15A94, Grass Instruments, West Warwick, RI, USA) configured to band-pass filter signals between (3 and 1000) Hz and digitalize them at a rate of 10 kHz with 16 bits.

To evaluate voluntary and involuntary PFM activity, clinicians designed a protocol of voluntary contractions that was performed by the subjects during each sEMG recording session consisting of 5 maximum voluntary contractions of 5 s each separated by 10 s of maximum relaxation. The signals were recorded from 2 min before the first contraction to 2 min after the last.

### 2.3. sEMG Signal Conditioning

sEMG recordings were off-line processed in MATLAB studio (version R2018b). A bipolar signal from each PFM side was obtained from monopolar recordings: B1 = M1 − M3 (right side) and B2 = M2 − M4 (left side). Spectral components of sEMG monopolar and bipolar signals outside the frequency range of the PFM activity were removed with two filters applied consecutively and symmetrically: a 17th order Butterworth low-pass filter (cut-off frequency: 450 Hz) and an 8th order Butterworth high-pass filter (cut-off frequency: 30 Hz). Such non-desired spectral components are related to motion artifacts and noise generated in the amplification system and at the skin-electrode interface, among others [34]. An additional comb filter was used to remove the power interference in the signal (50 Hz) and its harmonics. It was also applied symetrically to avoid displacing frequency components of the sEMG spectrum.

Different segments were mannually annotated in preprocessed sEMG signals acquired in each recording session to assess the voluntary and involuntary activity of the PFM:

(1) 5 segments of 5 s of single contractions, one for each maximum voluntary contraction performed by the PFM.
(2) 1 segment of 10 s of basal activity before the first contraction, when the PFM shows a maximum state of relaxation [35].
(3) 1 segment of 67 s from 1 s before the first contraction to 1 s after the last contraction, including the 5 contractions and the 4 resting periods.

Intermittent artifacts were excluded from the annotated segments.

### 2.4. sEMG Signal Parametrization

Temporal, spectral, and non-linear parameters were computed from annotated sEMG segments to characterize the PFM activity of healthy women and CPP patients: root mean square (RMS), median frequency (MDF), Dimitrov index (DI), sample entropy (SampEn), and normalized cross-correlation (CC).

RMS is a temporal parameter used to estimate the amplitude of the signal, consisting of the square root of the power of the signal [15]. The RMS of a discrete time series *x[n]* with *N* samples is calculated as follows:

(1) $$RMS=\sqrt{\frac{1}{N}\sum_{k=1}^{N}x{\left[k\right]}^{2}}$$

The amplitude of the sEMG signal is related to the number of muscle fiber action potentials [36]. Thus, a higher RMS value can be associated with a greater net activation of motor units, i.e., increased recruitment and/or discharge rates [20].

MDF is defined as the frequency that splits the power spectrum of the signal into two regions with the same total power. Considering *P(j)* as the value of the power spectrum at frequency *j* and *f_j_* as the signal sampling frequency, its MDF can be expressed according to the following formulation:

(2) $$\sum_{j=0}^{MDF}P(j)=\sum_{j=MDF}^{f_{s}/2}P(j)=\frac{1}{2}\sum_{j=0}^{f_{s}/2}P(j)$$

Together with other estimators like the mean frequency or the dominant frequency of the power spectrum, MDF is a common index used to characterize the spectral content of sEMG signals. Variations in the value of MDF reflect modifications in the distribution of the signal power spectrum throughout its bandwidth, which can be associated with changes in the the conduction velocity of the action potential through muscle fibers and in the tissue filter effects, among other factors [37,38]. For this reason, MDF has been widely used to characterize changes in the spectral content of sEMG signals in the context of muscle force production and fatigue [37,39].

DI is a spectral parameter defined to assess peripheral muscle fatigue with a higher sensitivity than traditional spectral indexes [40] that has proved to be a robust estimator in dynamic conditions [41]. Its value is computed as the ratio between the spectral moment of order −1 and the spectral moment of order *k* (*k* = 2, 3, 4 or 5) of the signal [40,42]:

(3) $$DI=\frac{\sum_{j=0}^{f_{s}/2}j^{-1}\cdot P(j)}{\sum_{j=0}^{f_{s}/2}j^{k}\cdot P(j)}$$

The numerator and denominator of this expression emphasize low and high frequency components of the signal, respectively [40], so that greater DI values are associated with increased peripheral fatigue. In the present study, *k* = 5 was selected for the superior performance of this value with respect to the others [42].

SampEn is an estimation of the irregularity or level of complexity of the signal in the temporal domain. It is computed as the negative of the natural logarithm of the conditional probability that two numerical series of *N* samples that are alike at *m* points (probability: *B^m^(r)*) remain similar at *m* + 1 points, according to a tolerance of *r* and not considering matches of subsequence in the numerical series with itself [43]. SampEn is formulated as follows:

(4) $$SampEn(N,m,r)=-\log\frac{A^{m}(r)}{B^{m}(r)}$$

where *B^m^(r)* is the probability of similarity at m points and *A^m^(r)* at *m* + 1 points. In the present work, a Z-score normalization was performed on sEMG signals before computing their SampEn. Subsequences of *m* = 2 samples were compared under a tolerance of *r* = 0.15. SampEn has been used to characterize numerical series in quite diverse contexts like the analysis of heart rate variability in neonates and patients with obstructive sleep apnea syndrome from electrocardiographic signals [44,45] or for emotion recognition from electroencephalographic recordings [46].

*CC* is a temporal parameter used to study interactions between two sEMG signals [47]. For two numerical series (*x*[*n*], *y*[*n*]) delayed *τ* samples, *CC* can be calculated as follows:

(5) $$CC(\tau )=\frac{E[(x[n]-\bar{x}){\left(y[n+\tau ]-\bar{y}\right)}^{*}]}{\sigma_{x}\sigma_{y}}$$

where *E* is the expected value, * the conjugate transpose, $\bar{x}$ and $\bar{y}$ the mean values of *x*[*n*] and *y*[*n*], respectively, and *σ_x_* and *σ_y_* their standard deviations. When *CC* is computed from sEMG signals of two different muscles or regions in the same muscle, *CC* provides an estimation of the synchronization of their motor units [20]. Its absolute value ranges from 0 to 1, so values near to 1 are associated with a greater level of synchronization.

For each session of recording, RMS, MDF, DI and SampEn were computed from segments of single contractions and relaxations annotated in bipolar signals (B1, and B2). Values obtained from the 5 contractions were averaged, so that PFM voluntary activity was assessed according to a single value per parameter in each recording session. *CC* was computed from one segment of the signal that included the five contractions performed by patients and the relaxing periods between them. Specifically, *CC* was calculated between segments of channels M1 and M3 (right side) and between segments of channels M2 and M4 (left side).

### 2.5. Statistical Analysis

The influence of the group (CPP, >35/P, and <35&NP) on the characteristics of the pelvic sEMG signal was assessed, giving significant differences in the distribution of values between the groups for each PFM side and sEMG parameter. According to a two-tailed Kolmogorov-Smirnov test (significance level: 5%), the data did not follow a normal distribution, so a two-tailed Kruskal–Wallis test was performed under 5% of significance. A post hoc analysis based on the Bonferroni method was carried out to determine the pair of groups that showed significant differences in their sEMG characteristics.

## 3. Results

**Figure 2:**
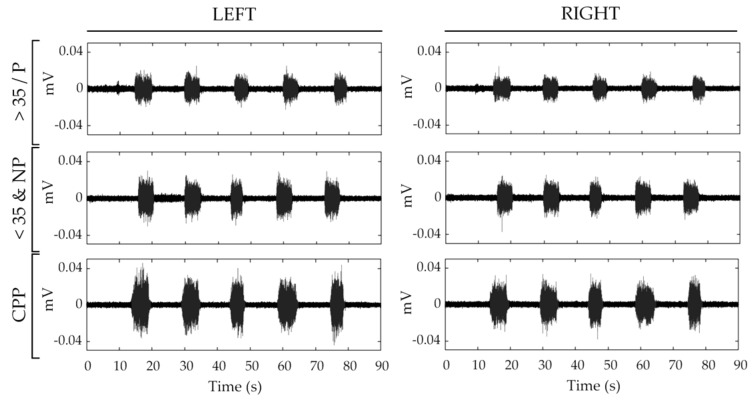
sEMG signals recorded from the pelvic floor musculature (PFM) of a subject from each group: woman over 35 and/or parous (>35/P, first row), woman under 35 and nulliparous (<35&NP, second row) and woman with Chronic pelvic pain (CPP, third row). Left and right subfigures show signals recorded from each subject’s left and right PFM, respectively.

Figure 2 displays the sEMG signals recorded during the protocol of contractions from the left and right sides of the PFM from a subject in each group. It can be seen that the signal amplitude was highest in the woman from the CPP group and lowest in the woman from the >35/P group.

**Figure 3:**
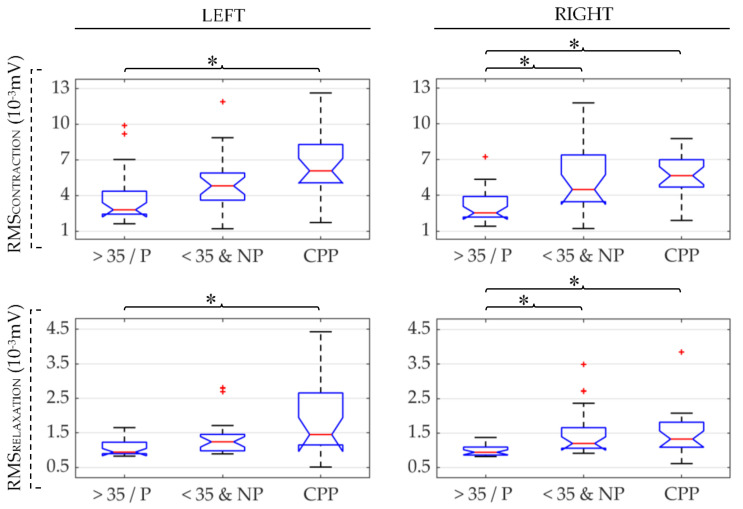
Box-whisker plots of root mean square (RMS) values of the left and right PFM (left and right subfigures, respectively) in patients (CPP), healthy women over 35 and/or parous (>35/P) and healthy women under 35 and nulliparous (<35&NP) during PFM contraction and relaxation (first and second rows, respectively). (*): significant difference between groups under the ends of the brace. Red (+): outlier within the group’s data distribution.

**Figure 4:**
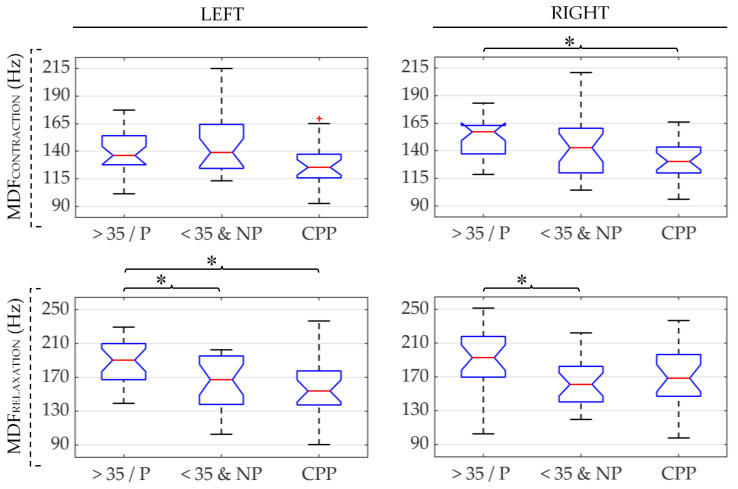
Box-whisker plots of MDF values of the left and right PFM (left and right subfigures, respectively) in patients (CPP), healthy women over 35 and/or parous (>35/P) and healthy women under 35 and nulliparous (<35&NP) during PFM contraction and relaxation (first and second rows, respectively). (*): significant difference between groups under the ends of the brace. Red (+): outlier within the group’s data distribution.

**Figure 5:**
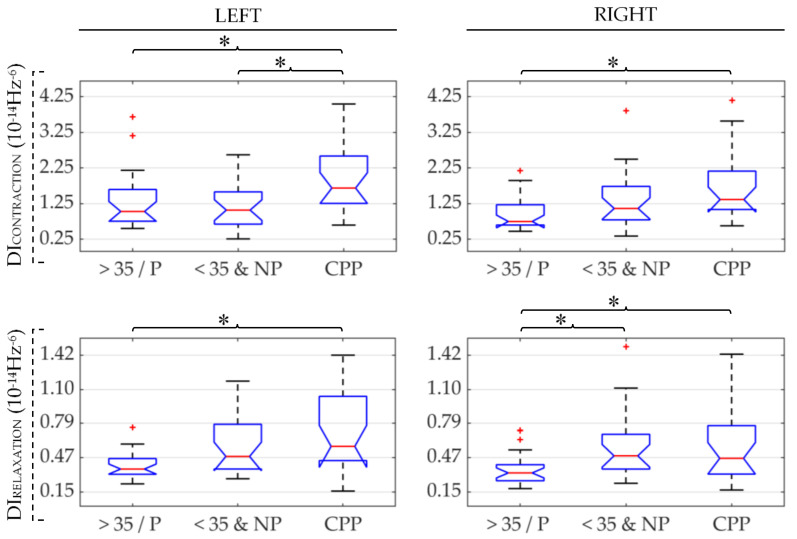
Box-whisker plots of Dimitrov index (DI) values of the left and right PFM (left and right subfigures, respectively) in patients (CPP), healthy women over 35 and/or parous (>35/P) and healthy women under 35 and nulliparous (<35&NP) during PFM contraction and relaxation (first and second rows, respectively). (*): significant difference between groups under the ends of the brace. Red (+): outlier within the group’s data distribution.

**Figure 6:**
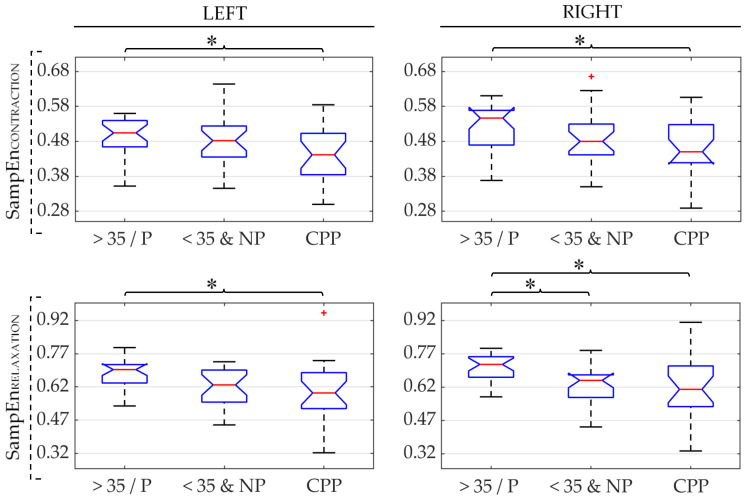
Box-whisker plots of sample entropy (SampEn) values of the left and right PFM (left and right subfigures, respectively) in patients (CPP), healthy women over 35 and/or parous (>35/P) and healthy women under 35 and nulliparous (<35&NP) during PFM contraction and relaxation (first and second rows, respectively). (*): significant difference between groups under the ends of the brace. Red (+): outlier within the group’s data distribution.

Figure 3 shows box-whisker plots of RMS values obtained from sEMG signals of the three groups under study (CPP, >35/P, and <35&NP). The results obtained for the left and right sides of the PFM are displayed in the left-hand and right-hand subplots, respectively. RMS values computed during the PFM contraction are depicted on the upper panels and those obtained during PFM relaxation are shown on the lower. In each subplot, an asterisk (*) has been used to indicate a significant difference in the distribution of RMS of those groups under the ends of the brace. The same panel layout and symbols are used to represent the results obtained for MDF, DI, and SampEn in Figure 4, Figure 5 and Figure 6, respectively.

It can be seen in Figure 3 that median RMS values from CPP were the highest, followed by <35&NP and >35/P, respectively. Regardless of the state of activation (contraction/relaxation), significant differences were found on both sides of the PFM in the comparison of CPP vs. >35/P, while differences between CPP vs. <35&NP were not significant. Statistical comparison of healthy groups showed that the RMS values from <35&NP group were significantly higher in the right side of the PFM both during contraction and relaxation.

As Figure 4 shows, smaller MDF values were usually obtained for the CPP group, followed by <35&NP and >35/P, respectively. Nonetheless, the differences between the pathological and healthy groups were only statistically significant on the left side during PFM relaxation and on the right during contraction when compared with the >35/P group. The comparison between the healthy groups showed significantly higher MDF values for the >35/P group on both PFM sides at rest.

The results of DI, shown in Figure 5, showed a similar trend to that of RMS, i.e., the highest median values for CPP, followed by <35&NP and >35/P, respectively. DI in CPP patients was significantly higher than in their healthy counterparts (>35/P group) on both PFM sides during contractions and relaxation. Differences in CPP with the younger healthy group (<35&NP) were significant only on the left side during PFM contraction. Differences between >35/P and <35&NP groups were significant only on the right when PFM was relaxed.

Figure 6 depicts the results of SampEn. It can be seen that the median SampEn values were the smallest in the CPP group. Significant differences between CPP and >35/P groups were found in the same way as for the RMS and DI parameters: on both PFM sides and contractile states (contraction and rest). Conversely, differences between CPP and <35&NP groups were not statistically significant in any case. In the comparison of healthy groups, differences were statistically significant only on the right PFM during relaxation.

**Figure 7:**
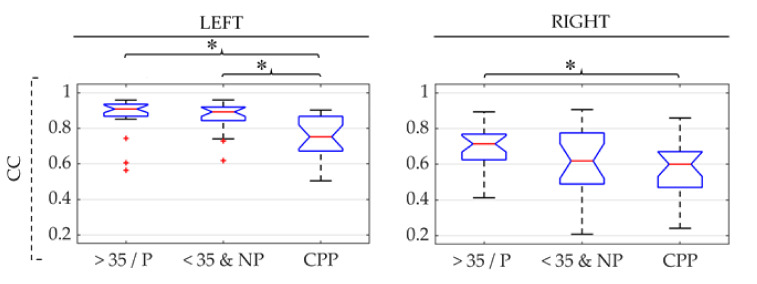
Box-whisker plots of cross-correlation (CC) values between the upper and lower sides of the left and right PFM (left and right subfigures, respectively) in patients (CPP), healthy women over 35 and/or parous (>35/P) and healthy women under 35 and nulliparous (<35&NP) during the five PFM contractions and relaxations between them (first and second rows, respectively). (*): significant difference between groups under the ends of the brace. Red (+): outlier within the group’s data distribution.

Box-whisker plots of the CC values obtained from sEMG signals on each PFM side of the three groups are shown in Figure 7. These results are the CC values calculated without considering delays between the two signals under study. They were almost equal to maximum CC values, which were obtained at lags of 0.2 ms (2 samples) at most in all groups. It can be seen in Figure 7 that median CC was lowest for CPP, followed by <35&NP and >35/P, respectively. Differences between pathological and healthy groups were significant on both sides of the PFM for >35/P and only on the left for <35&NP. No significant differences were found between healthy groups. It is noteworthy that CC values were greater on the left than on the right in the three groups.

Table 2 gives a summary of the significant differences obtained between sEMG characteristics of the three groups under study with the significantly different parameters in the two groups. A vertical arrow indicates higher values in the first group (↑) or in the second (↓). Most of the significant differences were found between CPP and >35/P groups on both sides of the PFM in nearly all the sEMG characteristics. Conversely, almost no significant difference was obtained when the pathological group was compared with <35&NP subjects. In the comparison between >35/P vs. <35&NP significant differences were found mainly on the right PFM at rest.

## 4. Discussion

In this study, the PFM activity of women with different clinical characteristics was analyzed: patients with CPP associated with deep dyspareunia, healthy women over 35 (“mature”) and/or parous and healthy women under 35 (“young”) and nulliparous. Unlike most studies in the literature, the assessment was not limited to the analysis of the power of the PFM activity, but included other characteristics such as its spectral content, complexity or synchronization between different regions of the muscle to achieve a better understanding of the PFM electrophysiological condition.

The RMS of the sEMG signal was computed to characterize the power of the PFM activity. Its value was higher in patients than in healthy women during maximum contractions and relaxation, especially when compared with mature/parous healthy women. Like us, several other authors have observed an increased amplitude of the sEMG signal in muscle at rest in women with PFM-associated pelvic pain [16,17,48], which has traditionally been associated with an increased level of neural excitation [13]. However, unlike us, some authors have reported no differences in the amplitude of the signal during PFM contractions [18,49] or even lower values [16,17] when the patients were compared with healthy subjects. As Gentilcore-Saulnier et al. [50] have pointed out, intracavitary probes were used in those studies to record PFM activity in patients, so the pain experienced during voluntary activation of the PFM could have prevented them from obtaining the maximum contraction. In addition, in these studies the painful symptoms were mainly associated with the vulva and the bladder, while in our work they were mostly associated with the deep vaginal region, so that different alterations of the PFM electrophysiological condition and function could be expected, although further research is needed to clarify this question.

Regarding the power of the PFM activity of healthy subjects, we obtained higher values for younger-nulliparous women during PFM contractions and relaxation, especially on the right side. Pereira et al. [24] observed that the amplitude of the sEMG signal was negatively correlated with age and parity, while Petricelli et al. [27] reported that it was significantly higher in pregnant nulliparous than in multiparous women. Although their assessments were limited to voluntary PFM activation and no distinction was made between PFM sides in either study, their results are along the same lines as ours. Castro-Pardiñas et al. [26] also obtained greater mean RMS values in nulliparous healthy than in multiparous women and in women less than 45-years old than in older subjects, though these differences were not statistically significant. They also analyzed sEMG signals only during PFM maximum contractions. However, they did measure the basal tone of the muscle by manometric probes and found that it was significantly higher in nulliparous and young women. Since the basal tone of the PFM is a product of its tonic myoelectrical activity [51], it can be assumed that this result agrees with the differences obtained in the RMS at-rest values of both healthy groups in the present work. Some studies found that aging and parity involve denervation of the PFM [52,53], so that the inability to recruit some motor units would account for the lower PFM myoelectrical activity power we found in mature/parous healthy women than in young nulliparous healthy women.

MDF values computed from sEMG recordings of CPP patients were lower than those from healthy women, significantly with those from mature/parous healthy women, and their DI values were higher. These differences reflect a shift of the sEMG power spectrum towards lower frequencies, which is usually associated with a reduction of the average conduction velocity of motor units. The lower conduction velocity of the action potential is a characteristic feature in peripheral fatigue [21]. Considering that some works have reported a state of fatigue in overactive muscles [54], this condition could probably account for significant differences in the sEMG spectral content of patients compared with their healthy counterparts. The electrophysiological processes underlying differences in the spectral parameters of young nulliparous and mature/parous healthy women are not so evident. As previously mentioned, variations in MDF and especially in DI have traditionally been related to the reduced conduction velocity of motor units, and thus to the development of peripheral fatigue. However, changes in the spectral content of the sEMG signal can also be triggered by alterations of other factors [37]. Different authors have reported morphological changes in the female pelvic floor with ageing [55,56,57], including a significant transition of PFM shape from V-like to U-like. Alterations in PFM shape or morphology have also been found in the case of parity [58,59]. The differences between the spectral content of both healthy groups could thus be due to a modification of the spatial filtering of their sEMG signals due to changes in the volume conductor effects [20]. In a similar direction as our study, Quartly et al. [60] reported a positive relationship between age and PFM endurance, measured by intravaginal pressure probe, during sustained contractions. They attributed this to the increased proportion of Type I (fatigue resistant) muscle fibers that various authors had previously associated with aging [61]. However, a study carried out by Dimpfl et al. [62] on female cadavers of different ages and parity revealed that the proportion of Type I and II muscle fibers in the levator ani muscles was no different between groups.

SampEn values were smaller in CPP patients than in healthy groups, especially in mature/parous women. Fatigue could once more account for the higher levels of organization found in the PFM activity of CPP patients. As several authors have suggested [22], the recruitment of motor units within a given muscle region could be more synchronized in conditions of fatigue and would lead to increased determinism in the sEMG signal and consequently to lower complexity values. In the comparison between mature/parous and young and nulliparous healthy women, the lower complexity observed in the PFM activity of the latter group disagrees with the widespread assumption that the determinism or regularity in aging systems is greater than in healthy ones [63]. Nevertheless, some studies have proved that this general premise may not always be true, depending on the physiological process or anatomic system under study. In particular, Kang and Dingwell [64] observed that the complexity of the activity decreased with age in proximal leg muscles, while it increased in the gastrocnemius. As the neural drive signal to this muscle is lower in adults, they concluded that an increase of the relative power of white noise in the signal could account for this finding. As explained above, age and parity are positively correlated with PFM denervation [52], so that a reduced sEMG signal-to-noise ratio, already quite low in at-rest PFM, could explain why the complexity of this activity was higher in mature/parous healthy women than in young and nulliparous healthy women in the present study. On the other hand, the variability of muscle fiber diameters and the degree of fibrosis increase significantly with parity and age [58,62]. These and other non-reported histomorphological changes could involve variations in the PFM activation pattern and thus play a role in the different complexity values found between healthy groups.

CC values were lower in patients than in healthy women in both groups. Some authors have previously pointed that the coordination between both PFM sides can be altered in pelvic floor disorders [65]. Conversely, our results suggest an impaired synchronization of two regions within the same hemipelvis in cases of CPP in which the PFM has a main role in its pathophysiology. This lower synchronization could be attributed to an altered propagation of the action potential throughout the same PFM side. However, no significant differences were found in delays between signals from the same side among the three groups. On the other hand, we found that median CC values of the left side were higher than those of the right in the three groups under study. Although it was beyond the scope of this study, the statistical significance of this difference was also assessed according to the Wilcoxon signed rank test, and *p*-values lower than 0.001 were obtained in all cases (results not shown). These results suggest that the rate of synchronization within the same side of the muscle is higher in the left than right PFM. To the best of our knowledge, the contrast between the electrophysiological behavior of each side of the PFM has not been previously reported in the literature. Differences in the density of innervations have rather been found between both sides of the PFM, specifically in the anal sphincter, as well as in the contribution of pudendal and levatorani nerves to the innervation of each of these [66,67,68]. However, Zacesta et al. [69] observed that the right side had a greater number of innervation zones in half of the cases and the left side in the other half in women both during pregnancy and after delivery, while Wallner et al. [68] reported that differences between the nerve supply on each side varied between subjects rather than following a given trend in a study performed on human fetuses. According to the existing studies in the literature, differences between the innervation of each PFM side would therefore not explain why all women showed higher CC values on the left PFM in the present study. When raw sEMG signals were visually inspected after their acquisition, electrocardiogram (ECG) interference was identified in many of them. Considering that the ECG power is greater on the left than on the right side of the trunk, due to the anatomical position of the heart [15], its presence in the sEMG signal could lead to higher CC values on the left PFM. Nevertheless, sEMG recordings were band-pass filtered between 30–450 Hz and most of the ECG power is concentrated between 0 and 40 Hz [70], so that its relative power in pre-processed signals should be minimal and thus its influence on the CC values obtained. On the other hand, when we compared RMS values on each side of the PFM, we found that they were significantly higher on the left than in the right in CPP patients and mature/parous healthy women (results not shown). Hence, the greater relative power of noise in monopolar signals recorded on the right PFM could be associated with the lower CC values obtained between them. However, similar RMS values were obtained on each PFM side in the case of young and nulliparous healthy women, while the left-hand CC was still significantly higher, so that deeper research is needed to elucidate the reason for this finding.

To sum up, differences between CPP patients and their healthy counterparts (mature/parous group) were significant for almost all parameters during the contraction and relaxation of both muscle sides, while differences between CPP and healthy, young, and nulliparous subjects were only significant for two parameters and only on the left side. The discrepancies in the results obtained from both PFM sides were even more marked in healthy groups: while all sEMG characteristics of the right PFM at muscle rest were significantly different between the groups, there were almost no differences in the activity on their left sides. Although the PFM is regarded as a functional unit whose sides cannot be activated independently, they are innervated by different branches of the same nerve [68], so that variations in their myoelectrical activity are possible [65,71]. In the case of healthy groups, the differences between the results on each side could be related to the fact that steeper myogenic changes take place with age and parity on the right than left PFM side [72].

The impact of laterality of pain on sEMG characteristics was analyzed in some preliminary studies (results not shown), but no significant relationships were found. Considering possible differences between the electrophysiological activity of both PFM sides, data were separated into different groups according to the patients’ laterality of pain (left side, right side, or both sides), so that the absence of significant relationships could be a consequence of the small sample size of the study. On the other hand, the pathophysiology of chronic pelvic pain involves multiple and complex mechanisms associated with somatic or visceral tissues (local/peripheral mechanisms) and the central nervous system (central mechanisms) [73]. Thus, although pelvic floor muscles can play a main role in the etiology of chronic pelvic pain, the association of pain with a given PFM side could be highly influenced by other factors, therefore there may not necessarily exist any direct relationship between the laterality of pain and the rate of alterations shown by sEMG characteristics.

One of the main challenges of the present study was the recruitment of healthy women with similar demographic and obstetric characteristics to those of patients. Because of the lack of volunteers, we finally decided to compare the PFM activity of patients with that of women who met at least one of two requirements: (1) over 35 years old, (2) parous. Although aging, pregnancy and delivery are associated with significant changes in the pelvic floor’s anatomy and functionality [58,59,62], these variations are different in the three physiological processes. A higher number of healthy women with different demographic and obstetric characteristics should be recruited to form more specific groups and individually assess the impact of each of these conditions on PFM activity.

## 5. Conclusions

PFM activity was assessed in women with CPP associated with deep dyspareunia and healthy women with different demographic and obstetric characteristics from sEMG recordings. Unlike the vast majority of previous studies in this field, a more comprehensive characterization was made of the PFM activity in both physiological and pathological conditions. Not only power but also spectral content and complexity of the PFM activity were evaluated, as well as the synchronization of different regions on the same PFM side.

The power of the PFM activity was greater in patients than in healthy women, especially when compared with mature/parous subjects, while their spectral content was shifted towards lower frequencies and complexity was lower. The synchronization of the lower and upper regions on the same PFM side was also lower in patients than in healthy women. These differences could be due to an increased number of motor units recruited and the muscle fatigue caused by overactivation of the PFM. In healthy conditions, the same trend of differences was observed when the PFM activity of young nulliparous women was compared with that of mature and/or parous subjects, which could be explained by histomorphological and neural changes associated with aging and parity.

The significant differences found between PFM activity in patients and their healthy counterparts prove that sEMG can be a useful tool for CPP clinical management, as it can provide specialists with objective information on the electrophysiological PFM conditions, the role of musculoskeletal pelvic structures in CPP pathophysiology and lead to better diagnosis and efficient management of patients.

## Figures and Tables

**Table 1:** Demographic and obstetric characteristics of women from groups Chronic pelvic pain (CPP), over 35 and/or parous (>35/P) and under 35 and nulliparous (<35&NP). Symbols show statistically significant differences between each pair of groups: (*) for CPP vs. <35&NP and (◊) for >35/P vs. <35&NP.

		CPP	>35/P	<35&NP
Age	mean ± SD (y)	43.8 ± 8.8 *	40.9 ± 7.2 ◊	28.1 ±3.2 *◊
Weight	mean ± SD (kg)	65.8 ± 11.2 *	61.8 ± 9.3	57.1 ± 7.3 *
Height	mean ± SD (cm)	162.6 ± 5.4	162.0 ± 3.8 ◊	165.8 ± 6.2 ◊
No. deliveries	mean ± SD	1.5 ± 0.7 *	1.5 ± 0.8 ◊	0.0 ± 0.0 *◊
Menopause	N (%)	8 (33.3%) *	3 (12.5%)	0 (0.0%) *
Laterality of pain	N (%)	4 (16.6%) Right 10 (41.7%) Left 10 (41.7%) Bilateral		

**Table 2:** Summary of differences between the sEMG characteristics of patients (CPP), healthy women over 35 and/or parous (>35/P), and under 35 and nulliparous (<35&NP). Results of PFM left and right sides during contraction and relaxation. Cells show the parameters with a significant difference between groups. Arrows ↑ or ↓ are displayed to indicate higher or lower value in the first than in the second group.

	CPP vs. >35/P		CPP vs. <35&NP		>35/P vs. <35&NP	
	Left	Right	Left	Right	Left	Right
Contraction	RMS ↑	RMS ↑				RMS ↓
		MDF ↓				
	DI ↑	DI ↑	DI ↑			
	SampEn ↓	SampEn ↓				
Relaxation	RMS ↑	RMS ↑				RMS ↓
	MDF ↓				MDF ↑	MDF ↑
	DI ↑	DI ↑				DI ↓
	SampEn ↓	SampEn ↓				SampEn ↑
Contractions and relaxations	CC ↓	CC ↓	CC ↓			

## Data Availability

The data are not publicly available since subjects enrolled in the study were not explicitly asked whether they consented to the sharing of their data.
